# Parkinsonian Symptoms, Not Dyskinesia, Negatively Affect Active Life Participation of Dyskinetic Patients with Parkinson’s Disease

**DOI:** 10.5334/tohm.403

**Published:** 2020-07-08

**Authors:** Etienne Goubault, Sarah Bogard, Pierre J. Blanchet, Erwan Bézard, Claude Vincent, Davide Martino, Justyna Sarna, Oury Monchi, Christian Duval

**Affiliations:** 1 Laboratoire de Simulation et Modélisation du Mouvement,École de Kinésiologie et des sciences de l’activité physique, Université de Montréal, Rue Jacques-Tétreault, Laval, Québec, CA; 2 Département des Sciences de l’activité physique, Université du Québec à Montréal, Montréal, Québec, CA; 3 Centre de Recherche de l’Institut universitaire de gériatrie de Montréal, Montréal, Québec, CA; 4 Département de stomatologie, Faculté de médecine dentaire, Université de Montréal, Montréal, Québec, CA; 5 Département de médecine, CHU Montréal, Montréal, QC, CA; 6 Université de Bordeaux, Institut des Maladies Neurodégénératives, Bordeaux, FR; 7 Centre National de la Recherche Scientifique Unité Mixte de Recherche, Institut des Maladies Neurodégénératives, Bordeaux, FR; 8 Département de réadaptation, Faculté de médecine, Université Laval, Québec, Québec, CA; 9 Cumming School of Medicine, Department of Clinical Neurosciences, University of Calgary, CA; 10 Hotchkiss Brain Institute, Department of Clinical Neurosciences, University of Calgary, Calgary, Alberta, CA

**Keywords:** Parkinson’s disease, Levodopa-induced dyskinesia, Participation, Every-day life, Activity engagement

## Abstract

**Background:**

The impact of slight-to-moderate levodopa-induced dyskinesia (LID) on the level of participation in active life in patients with Parkinson’s disease (PD) has never been objectively determined.

**Methods:**

Levels of LID, tremor and bradykinesia were measured during best-ON state in 121 patients diagnosed with PD and having peak-dose LID using inertial sensors positioned on each body limb. Rigidity and postural instability were assessed using clinical evaluations. Cognition and depression were assessed using the MMSE and the GDS-15. Participation in active life was assessed in patients and in 69 healthy controls using the Activity Card Sort (ACS), which measures levels of activity engagement and activities affected by the symptomatology. Outcome measures were compared between patients and controls using ANCOVA, controlling for age or Wilcoxon-Mann-Whitney tests. Spearman correlations and multivariate analyses were then performed between symptomatology and ACS scores.

**Results:**

Patients had significantly lower activity engagement than controls and had significantly affected activities. LID was neither associated with activity engagement nor affected activities. Higher levels of tremor, postural instability, cognitive decline and depression were associated with lower activity engagement and higher affected activities. Multivariate analyses revealed that only tremor, postural instability and depression accounted significantly in the variances of these variables.

**Discussion:**

Slight-to-moderate LID had little impact compared to other symptoms on the level of participation in active life, suggesting that other symptoms should remain the treatment priority to maintain the level of participation of patients in an active lifestyle.

## Introduction

We recently demonstrated, using a quantitative approach, that residual symptoms of Parkinson’s disease (PD) can coexist with levodopa-induced dyskinesia (LID), even at peak-dose [1]. In addition, we showed that slight-to-moderate LID had little influence on the motor performance of patients executing specific activities of daily living (ADL) [2]. Instead, we found that levels of tremor, postural instability, rigidity, and cognitive decline increased the odds of failure, depending on the tasks performed. While this study provided valuable information on the impact of symptomatology during specific movements, it did not provide an estimation of the impact of LID or other symptoms on the level of overall participation in an active life. For example, in our previous study, walking, picking-up an object on a table or off the floor, or counting money were examined separately. However, each of those ADL could be part of a broader activity such as doing grocery shopping, gardening, etc. Accordingly, the impact of LID on the level of participation in active life of patients with PD remains unclear. To our knowledge, only Duncan and Earhart [3] investigated which factors are most related to proper levels of participation. To do so, they examined 62 patients with PD using the Activity Card Sort (ACS). Their results suggested that risk of falls and freezing of gait were the main predictors of decreased levels of participation in every-day life. Their study however examined patients at different stages of disease, not specifically on those presenting with LID.

Therefore, the present study aimed at revisiting the patients’ symptomatology detected in our previous study [2], and at determining their influence on the level of participation in active life through their levels of *activity engagement*, and *affected activities* measured by the ACS. The ACS was designed to record the *activity participation* of adults in instrumental, leisure and social activities. It provides an occupational profile of the types of activities the patient is engaged in, or has given up. As a research tool, it reports a person’s previous activities (before an illness or injury) and current activities and allows investigators to examine the specific activities affected by an illness, injury or change in health status. The ACS has been used as an outcome measure in studies exploring the impact of programs specifically designed for people with PD [4,5] and has been translated in several languages and adapted to different international cultures [6]. We hypothesized that a higher level of LID would not be associated with a decrease of participation in active life [7]. Finally, we took this opportunity to revisit the relationship between symptomatology and quality of life. Instead of using self-reported assessment of LID, we used the LID severity measured by our sensors at peak dose, which is not sensitive to anosognosia. We hypothesized that LID would not be a determining factor in quality of life, as suggested in previous studies [8,9,10,11].

## Methods

A correlational cross-sectional study was performed.

### Participants

One hundred and twenty-one patients diagnosed with idiopathic PD according to the UK Parkinson’s Disease Society Brain Bank clinical diagnosis criteria [12] were recruited with the help of the Quebec Parkinson Network (QPN), and clinicians specialized in movement disorders. Data collection was conducted in three Canadian research institutes: the Centre de Recherche de l’Institut universitaire de gériatrie de Montréal (CRIUGM), the Center of interdisciplinary research in rehabilitation and social integration (CIRRIS) of Quebec City, and the Movement Disorders Program of the University of Calgary, from June 2016 until June 2017. In this manuscript, data from patients originated from two others studies on the concomitant presence of LID and cardinal motor symptomatology [1], and on the impact of LID on the motor performance in ADL [2]. Motion capture methodology is detailed elsewhere [1,2]. In brief, patients selected in this study have experienced clinically-detectable choreic-type LID in the past months prior to the experiment. Patients requiring assistance to walk or having orthopedic conditions that could interfere with the performance of required tasks were not considered for the study. Sixty-nine healthy control participants were also recruited through the Centre de Recherche de l’Institut universitaire de gériatrie de Montréal (CRIUGM), to provide a ‘normal’ baseline of behavior. To be included in the study, healthy control participants had to be between 45 and 85 years old, and able to walk without assistance. Exclusion criteria included any orthopedic condition that could interfere with the execution of ADL, being under the influence of any medication that could cause issues with concentration and involuntary movements, or being unable to understand the consent form or the instructions. The study protocol was approved by the Comité d’éthique de la recherche vieillissement-neuroimagerie of the Centre de Recherche de l’Institut universitaire de gériatrie de Montréal for data that were collected in Montreal (CER IUGM 13-14-022), by the Comité d’éthique de la recherche sectoriel en réadaptation et integration sociale for data that were collected in Quebec (MP-2016-510), and by the Conjoint Health Research Ethics Board of University of Calgary for data that were collected in Calgary (REB 16-2551). Informed consent was obtained in writing from all individual participants included in the study after making sure they were able to understand all information.

### Standard protocol or procedures

Patients’ visit to the laboratory was usually planned in the afternoon, coinciding with their scheduled medication dose deemed to generate the highest LID amplitude. They were instructed to take their medication at their usual time of day, often while filling the administrative requirements for the study participation, and the administration of questionnaires. Despite a variability among patients in medication regimen, patients were tested under their own medication regimen, individually determined by movement disorders neurologists. This allowed for a better representation of their true symptomatology at best-ON, peak-dose effect, which was the goal of our study.

### Questionnaires administration

Questionnaires were administered prior to the quantitative recording session, while the effect of medication was building-up. If choreic-type LID were noticed by the evaluators, questionnaires were put aside to begin data collection, and were resumed at the end of the session. The Mini-Mental State Examination (MMSE), which has a high discriminant validity for detection of any cognitive disorder [13,14], was used to assess cognitive decline [15], while the 15-item Geriatric Depression Scale (GDS-15) was used to assess depression [16].

The main outcome measure of the present study was the level of *activity engagement* and *affected activities* by patients’ symptomatology. This was measured by the Activity Card Sort (ACS) [17]. Participants were asked to sort cards depicting daily actions representing four domains; (1) *instrumental activity* (*IA*); (2) *low-demand leisure activity* (*LDLA*); (3) *high-demand leisure activity* (*HDLA*); and (4) *social activity* (*SA*). In our study, each participant sorted the cards into the following categories: (1) ‘never done’; (2) ‘continued’ to do despite the symptomatology; (3) ‘do less’ because of the symptomatology; (4) ‘given-up’ due to their symptoms; and (5) ‘new’ activity since the symptomatology appeared. This unique sorting methodology provided us with a quantifiable measure of *activity engagement* by calculating the number of activities currently performed by patients. The number of *affected activities* was calculated using ‘given-up’ and ‘do less’ activities.

As a secondary outcome, we also assessed patients’ health-related quality of life (*QoL*) using the 12-item Short Form health survey version 2 (SF-12v2). The SF12, widely used to assess *QoL* in PD [18,19,20], yields two scores: the *physical component summary* (*PCS*) and the *mental component summary* (*MCS*) [21,22].

### Symptomatology assessment

The method for symptomatology assessment is described in details elsewhere [1]. In brief, the data collection regarding the symptomatology began as soon as the investigators visually noticed choreic-type LID. The testing period proceeded in two blocks of tasks, during which investigators regularly asked patients for feedback about whether they felt that the medication reached their most efficient stage, based on their experience. The first block was dedicated to the assessment of motor symptoms of PD such as bradykinesia, postural instability, and rigidity. Bradykinesia was assessed using a rapid alternating movement (RAM) task [23,24,25] consisting in performing pronation-supination movements of the forearm with maximal rotational amplitude and maximal velocity for 10 sec. The outcome measure was the averaged maximal angular velocity of each cycle of pronation-supination movements [26,27,28,29]. Postural instability and rigidity were both assessed using the items 3.12 and item 3.3 of the MDS-UPDRS respectively, since efficient objective assessment of these symptoms is not yet available. These measures were repeated three times.

The second block, performed in between repetitions of block 1, was dedicated to the measurement of tremor and LID using a set of 17 inertial sensors, each located on a specific body part, enabling for full body motion capture. Using the embedded accelerometers and gyroscopes employed in each sensor, we were able to objectively quantify motion associated with involuntary movements. This technique was used by us and others in the past [2,30,31,32,33,34,35,36,37,38,39]. The measurement of tremor and LID were performed during sitting ADL (reading, cutting-eating, counting money, eating soup, taking medication, drinking water), selected from a comprehensive list of ADL scales and questionnaires specific for PD. The tremor level is referring in this study to all kinds of tremor (rest, postural or kinetic). It was measured using gyroscopes signals (x, y, z) recorded on hands and filtered between 3.5 and 7.5 Hz to remove the voluntary movements and the noise. Signals were detrended and segmented in 5 sec windows before calculating the power spectral densities in all directions. These components were summed to generate the total power spectral density. The dominant frequency was identified, and the power dispersion, representing the width of a frequency band containing 68% of total power centered around that dominant frequency was calculated. A narrow dispersion (i.e., below 2 Hz) is indicative of a dominant oscillation typically associated with abnormal tremor. If present, the amplitude of tremor was calculated by computing the power estimation within the frequency band identified by the power dispersion calculation. If the dispersion around the dominant frequency was more than 2 Hz, the spectrum was visually verified to ensure the absence of a sharp peak of tremor. The tremor level of each window was then summed to generate a tremor level for the entire task.

The levels of choreic-type LID [27,30,31,32,40] were also measured while performing sitting ADL, using all gyroscope signals of all sensors, except those positioned on the arms that were directly involved in voluntary movements. Here we chose to exclude these sensors since voluntary movements and LID are in the same frequency bandwidth. This technique represented a compromise between including all LID and having a lot of component of voluntary movements, and excluding some LID in our measure in excluding the majority of voluntary movements that could lead to erroneous interpretation of results. Because LID are defined as involuntary, irregular, purposeless, non-rhythmic, abrupt, rapid, unsustained movements that seem to flow from one part of the body to another, affecting the neck, the trunk, and the limbs [41], we think that eliminating some LID in excluding voluntary movement will still give a good approximation about the level of LID in the body. Signals from gyroscopes (x, y, z) were band-pass filtered between 0.5 and 4 Hz to remove the noise, and then detrended. The magnitudes of the coordinate vectors (x, y, z) were computed at each frame resulting in new time series. These new time series were then segmented in 1 sec windows and power spectral densities were computed on each window. The power dispersion around the dominant frequency was verified to ensure that dyskinesia was not being confounded with tremor, before calculating energy spectrums. The mean of energies was calculated for each segment of each window (3 sensors for each leg, 3 sensors for each arm, 4 sensors for the back, 1 sensor for the head). Then, for each segment, we calculated the 75^th^ percentile plus 1.5 inter quartile range threshold, and we considered each point above that threshold as a possible outlier resulting in a superfluous, isolated voluntary movement. Outliers were visually verified by video analysis and were reset to the highest value in the acceptable range only if a superfluous voluntary movement was seen. The mean of each 1 sec window of each segment was then calculated and the sum of segments was calculated, given an average energy value per second, per task.

This design enabled us to identify the profile of symptomatology at rest and during movement when patients were best-ON, i.e., when LID were predominant [42,43], to quantify their amplitude objectively, and to determine whether other symptoms were present, hence better reflecting the state of the patient during his or her daily lives. The entire recording session lasted approximately 1 hour.

### Statistical analyses

First, we identified patients who experienced LID during the experiment by comparing the maximum amplitude of each patient’s LID during movement with a threshold set using movements of healthy controls (mean + 1SD), giving a high level a sensitivity (91%) and specificity (87%). This first selection of patients having LID was compared with annotations written during the experiment and with video recording of testing sessions, and groups were manually readjusted to avoid false positive and false negative. For each ADL, trials where dyskinetic patients were best-ON were identified and used to average the severity levels of LID and of other PD symptomatology in order to reflect the clinical severity at the peak-dose time point. For each ADL, trials where dyskinetic patients were best-ON were identified and used to average the severity levels of LID and of other PD symptomatology in order to reflect the clinical severity at the peak-dose time point. This means that patients were assessed at both best-ON state and peak-dose effect, which normally coincide [42,44,45].

The *activity engagement*, as well as the *affected activities* were compared between groups using analyses of covariance (ANCOVA) with age entered as a covariate. Health related *QoL*, represented by the *physical component summary* and the *mental component summary* scores of the SF-12v2, was compared between groups using Wilcoxon-Mann-Whitney tests. Spearman correlations between recorded symptoms and *activity engagement, affected activities*, and health related *QoL*, were calculated in patients having LID with a Benjamini-Hochberg correction for p-values. Multiple regression analyses with backward elimination of factors were performed to understand which symptoms explain the variability of *activity engagement, affected activities*, and health related *QoL*. This statistical design included all patients, regardless of symptomatology present. This allowed us to determine whether specific symptoms were more or less deleterious than LID, when present. Analyses were done using R (R Foundation for Statistical Computing, Vienna, Austria). All statistical significance thresholds were set at α < 0.05.

## Results

From the one hundred and twenty-one patients with PD, three were excluded from the analysis; one presented with severe osteoarthritis, the two others did not complete the experiment because of severe postural instability. Eighty-eight (74.6%) of the remaining patients reached our threshold criterion of LID severity. Eighty of them had a slight-to-moderate LID, with a score ranging from 1 to 3 in the MDS-UPDRS item 4.2. The characteristics of selected participants are presented in Table 1.

**Table 1:** Characteristics of participants.

Characteristics	Controls (n = 69; 30 females)	Group 1 (n = 88; 41 females)

Age (yr): mean ± SD	68.1 ± 7.7	67.5 ± 8.7
MMSE (/30)	28.6 ± 1.5	27.3 ± 2.5
GDS-15 (/15)^b^	1.7 ± 2.0	3.7 ± 2.8
SF-12-PCS	53.1 ± 5.5	42.0 ± 9.3
SF-12-MCS	49.9 ± 7.2	47.6 ± 8.5
Years since diagnosis		10.4 ± 5.5
LEDD (mg)		1048.9 ± 520.0^a^
MDS-UPDRS part III ON^b^		
Speech		1.2 ± 1.0
Facial expression (3.2)		1.6 ± 1.0
Arms rigidity (3.3)^c^		0.7 ± 0.7
Legs rigidity (3.3)^c^		1.1 ± 0.8
Arising from chair (3.9)		0.4 ± 0.7
Gait (3.10)		1.1 ± 0.9
Freezing of gait (3.11)		0.3 ± 0.7
Postural stability (3.12)		1.2 ± 1.0
Posture (3.13)		0.8 ± 0.9
Bradykinesia (3.14)		1.0 ± 1.0
Postural tremor (3.15)^c^		0.4 ± 0.8
Rest tremor (3.17)^c^		0.2 ± 0.5
MDS-UPDRS part IV^b^		
Time spent with LID (4.1)		1.6 ± 0.9
Functional impact of LID (4.2)		1.7 ± 1.0
Hoehn and Yahr score ON^b^		2.3 ± 0.7

^a^ Missing data for 9 participants. ^b^ Higher score indicates worse functioning. ^c^ Score represent the mean of the left and right extremities. MDS-UPDRS = Movement Disorder Society-Unified Parkinson’s Disease Rating Scale; SD = Standard deviation, LEDD = Levodopa Equivalent Daily Dose.

### Activity engagement

Patients presented a significantly lower *activity engagement* compared to controls, except for *social activity* where patients having LID did not differ from controls (Table 2).

**Table 2:** Results of ANCOVA on *activity engagement* controlling for age.

	Mean (SD) C	Mean (SD) P	Statistics	C – P

***IA***	14.62 (2.45)	12.30 (3.32)	F(1,154) = 28.19	**p < 0.001**
***LDLA***	18.90 (4.72)	15.70 (4.24)	F(1,154) = 20.91	**p < 0.001**
***HDLA***	6.09 (2.91)	4.54 (2.53)	F(1,154) = 16.26	**p < 0.001**
***SA***	11.72 (2.95)	11.07 (3.03)	F(1,154) = 1.95	p = 0.164
***ACS Total***	51.32 (10.43)	43.61 (10.58)	F(1,154) = 22.95	**p < 0.001**

Bold values indicate statistically significant (α = 0.05); C = Controls; P = Patients; *IA = Instrumental activity; LDLA = Low-demand leisure activity; HDLA = High-demand leisure activity; SA = Social activity.*

The levels of tremor, postural instability as well as cognition and depression significantly correlated with *activity engagement* after Benjamini-Hochberg correction, as shown in Table 3. The level of LID had no significant relationship with *activity engagement*.

**Table 3:** Correlations between recorded symptomatology and *activity engagement*.

	LID	Bradykinesia	Tremor	Rigidity	Postural instability	Cognition	Depression

***IA***	–0.152	**0.329**	**–0.236**	–0.029	**–0.451**	**0.250**	**–0.323**
***LDLA***	–0.060	0.013	–0.198	0.080	**–0.261**	**0.396**	**–0.330**
***HDLA***	–0.131	0.041	–0.209	0.051	**–0.489**	**0.263**	**–0.279**
***SA***	–0.163	0.209	**–0.252**	0.035	–0.201	**0.296**	**–0.389**
***ACS Total***	–0.157	0.174	**–0.282**	0.050	**–0.395**	**0.386**	**–0.372**

Bold values indicate correlation values significantly different from zero after a Benjamini-Hochberg correction for p-values (α = 0.05); *IA = Instrumental activity; LDLA = Low-demand leisure activity; HDLA = High-demand leisure activity; SA = Social activity.*

The multiple regression analysis with backward elimination of factors included only three measures, depression (β = –5.04, p = 0.001), tremor (β = –3.73, p = 0.01), and postural instability (β = –3.38, p < 0.001), which, once combined, explained 34% of the variance in the total *activity engagement*.

### Affected activities

When compared with normal aging of controls, patients had significantly more *affected activities* due to their symptomatology in *instrumental activity, high-demand leisure activity*, and *social activity* sub-scores, and in the *ACS total* score (Table 4).

**Table 4:** *Affected activities* of patients compared to controls.

	Mean (SD) C	Mean (SD) P	Statistics	C – P

***IA***	1.36 (1.72)	2.78 (2.66)	F(1,154) = 18.58	**p < 0.001**
***LDLA***	1.76 (2.15)	2.15 (2.57)	F(1,154) = 0.54	p = 0.464
***HDLA***	1.42 (1.53)	2.33 (2.35)	F(1,154) = 7.62	**p = 0.006**
***SA***	1.25 (1.49)	1.96 (2.02)	F(1,154) = 5.51	**p = 0.020**
***ACS Total***	5.47 (5.71)	9.22 (8.02)	F(1,154) = 9.29	**p = 0.003**

Bold values indicate statistically significant differences (α = 0.05); C = Controls; P = Patients; *IA = Instrumental activity; LDLA = Low-demand leisure activity; HDLA = High-demand leisure activity; SA = Social activity.*

The level of LID was not significantly correlated with *affected activities*. However, significant correlations were found between *affected activities* and tremor, postural instability, and depression after Benjamini-Hochberg correction (Table 5).

**Table 5:** Correlations between recorded symptomatology and *affected activities*.

	LID	Bradykinesia	Tremor	Rigidity	Postural instability	Cognition	Depression

***IA***	0.188	–0.160	0.233	–0.100	**0.396**	–0.223	**0.262**
***LDLA***	0.137	–0.237	**0.257**	–0.104	**0.468**	–0.067	**0.327**
***HDLA***	0.216	–0.122	**0.266**	–0.074	**0.272**	–0.196	0.166
***SA***	0.217	–0.215	**0.349**	–0.020	**0.393**	–0.135	**0.352**
***ACS Total***	0.240	–0.207	**0.333**	–0.097	**0.448**	–0.193	**0.353**

Bold values indicate correlation values significantly different from zero after a Benjamini-Hochberg correction for p-values (α = 0.05); *IA = Instrumental activity; LDLA = Low-demand leisure activity; HDLA = High-demand leisure activity; SA = Social activity.*

The multiple regression analysis included the same three measures, depression (β = 0.44, p = 0.02), tremor (β = 0.45, p = 0.01), and postural instability (β = 0.53, p < 0.001), which, once combined, explained 36% of the variance in the total *affected activities*.

### Health related QoL

The *physical component summary*, and the *mental component summary* of patients were significantly poorer than controls (Table 6).

**Table 6:** Comparison of SF-12 scores between groups.

	Mean (SD) C	Mean (SD) P	Statistics	C – P

***PCS***	53.09 (5.49)	42.00 (9.26)	W = 5022	**p < 0.001**
***MCS***	49.92 (7.22)	47.60 (8.47)	W = 3533	**p = 0.042**

Bold values indicate statistically significant differences (α = 0.05); C = Controls; P = Patients; *PCS = physical component summary; MCS = mental component summary.*

The *physical component summary* score of the SF-12v2 was significantly correlated with levels of tremor, postural instability, cognition, and depression, while the *mental component summary* score was significantly correlated with depression after Benjamini-Hochberg correction (Table 7).

**Table 7:** Correlations between recorded symptomatology and health related *QoL*.

	LID	Bradykinesia	Tremor	Rigidity	Postural instability	Cognition	Depression

***PCS***	–0.204	0.136	**–0.296**	0.108	**–0.295**	**0.299**	**–0.294**
***MCS***	–0.193	0.124	0.097	0.083	–0.131	0.161	**–0.653**

Bold values indicate correlation values significantly different from zero after a Benjamini-Hochberg correction for p-values (α = 0.05); *PCS = physical component summary; MCS = mental component summary.*

The multiple regression analysis included also depression (β = –3.11, p < 0.04), tremor (β = –3.39, p = 0.02), and postural instability (β = –1.70, p < 0.08), which, once combined, explained 18% of the variance in the *physical component summary*. Regarding the *mental component summary*, depression (β = –7.70, p < 0.001), tremor (β = 2.38, p = 0.06), and dyskinesia (β = –1.10, p < 0.09) were retained in the final model, which combined explained 38% of the variance. It should be noticed that depression alone was able to explain 35% of the variance in *mental component summary*.

## Discussion

The results of this study demonstrate that patients with PD had significantly less *activity engagement* than healthy controls and had more *affected activities* due to their symptomatology, which was expected. Interestingly, the level of *activity engagement* was not associated with slight-to-moderate LID, but rather with other symptoms such as tremor, bradykinesia, postural instability, depressive symptoms and cognitive impairment. Similarly, the level of *affected activities* was not associated with slight-to-moderate LID, but rather with higher levels of tremor, postural instability, and depressive symptoms. A poorer *physical component summary* of the *QoL* was also associated with higher levels of tremor, postural instability, depressive symptoms, and cognitive impairment, while a poorer *mental component summary* was only associated with higher level of depressive symptoms.

Taken together with our previous work [2], our results contradict those suggesting that LID is responsible for reduced ADL, overall physical participation and QoL [46,47,48,49,50,51]. Indeed, we previously showed that slight-to-moderate LID was only problematic when the intended movement was of low amplitude and velocity and demanded accuracy (e.g., for eating soup). Conversely, LID increased the odds of success in some ADL by enhancing patients’ mobility during walking tasks [2]. The odds of success in ADL were rather decreased because of a) higher levels of tremor for other dexterous activities, b) postural instability for standing and walking ADL, and c) cognitive decline for activities requiring cognitive resources.

In the present study, the presence of slight-to-moderate LID was neither associated with reduced *activity engagement* nor increased *affected activities*, but the presence of other motor and non-motor symptoms of PD were associated with lower levels of these dependent variables. Specifically, cognition and depressive symptoms, reflected by the MMSE and GDS-15 scores, seemed to possess a stronger association with decreased perception of *activity engagement*, and increased *affected activities* of patients. The presence of slight-to-moderate LID was not associated with reduced *QoL*, while other motor and non-motor symptoms of PD were. This underlines the impact of residual motor and non-motor symptoms of PD on participation in every-day life and *QoL*, as alluded to by others [8,9,52,53,54,55,56,57].

In this study, the medication regimen of patients was not standardized and the visit was scheduled to coincide with their best-ON condition deemed to generate the most intense LID. We think that this state best reflects their true living condition when LID is present. The main strength of the present study comes from the objective assessment of motor symptoms. Previous studies mostly used questionnaires to assess the symptomatology and its relationship with outcome measures. The use of the UPDRS part IV is common to assess the presence and the severity of LID [58], which counteracts observational bias, and possible flaws in recall during self-assessment of symptoms [59,60]. A possible limitation, however, is that patients tested in our study only presented with slight-to-moderate LID. This could limit somewhat the scope of the results; it is reasonable to assume that severe LID may have a negative impact on every-day life participation, whereas slight-to-moderate LID may not [58,61].

## Clinical considerations

One strategy to alleviate peak-dose LID is to reduce the dosage of dopaminergic drugs, with the risk to compromise motor control [62,63,64]. Our previous results have highlighted the fact that symptoms of PD may be present simultaneously with LID [1], and that it is those symptoms that are detrimental to the performance of ADL. The present study highlights the same issues when broader activities are examined, as well as QoL. These results strongly suggests that the success of a change of medication to manage LID should not be solely measured by its ability to reduce LID, but also on its ability to maintain or enhance the motor repertoire of patients [65].

## Data Accessibility Statement

The datasets used and/or analyzed during the current study are available from the corresponding author on collaborative work.

## Abbreviations

ACS Activity Card Sort

ADL Activities of daily living

LID Levodopa-induced dyskinesia

GDS-15 15-item Geriatric Depression Scale

HDLA High-Demand Leisure Activity

IA Instrumental Activity

LDLA Low-Demand Leisure Activity

MCS Mental Component Summary

MMSE Mini-Mental State Examination

PCS Physical Component Summary

PD Parkinson’s disease

QoL Quality of life

RAM Rapid alternating movement

SA Social Activity

SF-12 12-item Short Form Survey
